# “We Follow the Disinformation”: Conceptualizing and Analyzing Fact-Checking Cultures Across Countries

**DOI:** 10.1177/19401612241270004

**Published:** 2024-08-11

**Authors:** Daniela Mahl, Jing Zeng, Mike S. Schäfer, Fernando Antonio Egert, Thaiane Oliveira

**Affiliations:** 1 Department of Communication and Media Research, University of Zurich, Zurich, Switzerland; 2 Instituto de Arte e Comunicação Social, Universidade Federal Fluminense, Niterói, Rio de Janeiro, Brazil

**Keywords:** fact-checking, misinformation, cross-national comparison, content analysis, automated content analysis, interviews

## Abstract

Democratic societies inherently depend on an informed citizenry. By shaping citizens’ voting behavior, fostering political cynicism, and reducing trust in institutions, misinformation can pose significant challenges to individuals and societies. Against this backdrop, fact-checking initiatives aimed at verifying the accuracy of publicly disseminated (mis)information have flourished worldwide. However, existing research is disproportionately oriented toward the Global North, with a focus on the United States and the most influential organizations. Equally scarce are comparative studies. To address these shortcomings, this study introduces a *context-sensitive framework* for analyzing fact-checking cultures and illustrates its application in a *cross-national comparative design* by contrasting two countries from the Global South and North: Brazil and Germany. Using a mixed-methods design, we integrate computational, qualitative, and quantitative content analysis of 11 fact-checking organizations and 13,498 fact-checking articles over 11 years (2013–2023), alongside qualitative semistructured interviews with fact-checkers (*N* = 10). Our findings reveal several areas of divergence and convergence, suggesting that fact-checking cultures transcend organizational and national boundaries.

## Introduction

Societal crises and upheavals—armed conflicts, migration, right-wing populism, economic turmoil, the climate crisis, and health emergencies—can contribute to perceived instability and uncertainty in societies (van Aelst et al. 2017). At such times, orchestrated misinformation campaigns often gain unprecedented momentum (Bennett and Livingston 2020), raising global concerns about their impact on public and political life (UNESCO 2023).

Misinformation can pose substantial challenges at the individual and societal levels, shaping citizens’ attitudes (van der Linden et al. 2017), decision-making (Kricorian et al. 2022), and voting behavior (Gunther et al. 2019), fostering political cynicism (Lee and Jones-Jang 2022), delegitimizing the press (Ognyanova et al. 2020), and eroding trust in political institutions (Boulianne and Humprecht 2023).

Fact-checking, aimed at verifying the accuracy of publicly disseminated claims, is often hailed as a vital antidote to misinformation (Miller and Vaccari 2020), even as a “democracy-building tool” (Amazeen 2020: 99). Consequently, fact-checking initiatives have recently thrived (Lowrey 2017), with 417 organizations operating in more than hundred countries (Stencel et al. 2023). In parallel, a burgeoning body of research has explored various facets of the fact-checking landscape (Graves 2018), its institutionalization (Singer 2021), fact-checker’s role perceptions (Brandtzaeg et al. 2018), practices (Coddington et al. 2014), work routines (Graves 2017), and the consistency of fact-checking in political debates (Lelo 2023). Moreover, the effects on political elites (Ceron and Carrara 2023) and the public (Fridkin et al. 2015), including citizens’ voting behavior and political knowledge (Barrera et al. 2020) and attitudes toward fact-checking (Lyons et al. 2020), have been studied. Considering the broader sociopolitical implications of misinformation, scholars have also extensively explored the relationship between political communication and fact-checking practices (Chia et al. 2022; Lyons et al. 2020; Robertson et al. 2020; Vinhas and Bastos 2023b; Walter et al. 2020).

Despite intensified research activity and the global upsurge of fact-checking, scholarship remains disproportionately oriented toward the Global North, with a focus on the United States (for overviews, cf. Dias and Sippitt 2020; Nieminen and Rapeli 2019), often examining the most influential organizations such as PolitiFact (Nieminen and Sankari 2021). This leaves scholars with an insufficient understanding of fact-checking outside the Global North and in non-English-speaking regions (exceptions are Cheruiyot and Ferrer-Conill 2018; Chia et al. 2022; Kumar 2024). Moreover, comparative studies, which are particularly suited to elucidate national specificities as well as transnational similarities, remain scarce (exceptions include Ferracioli et al. 2022; Humprecht 2020; Vinhas and Bastos 2023b).

Recognizing the need for comparative research on fact-checking efforts across countries with different media and political systems, journalistic cultures, and sociocultural contexts, this study offers a twofold contribution: First, it introduces a *context-sensitive framework* for analyzing fact-checking cultures. Second, it demonstrates the application of this framework in a *cross-national comparative design*, contrasting two countries from the Global South and North: Brazil and Germany. Employing a mixed-methods design, it integrates computational, qualitative, and quantitative content analysis of eleven fact-checking organizations and 13,498 fact-checking articles over eleven years (2013–2023), alongside qualitative semistructured interviews with fact-checkers.

## The Global Fact-Checking Movement

In contrast to “internal” fact-checking, a core journalistic practice which seeks to rectify errors before publication (Graves and Amazeen 2019), “external” fact-checking conducts “evidence-based assessments” (Graves et al. 2023: 2) of the veracity of statements already circulating in the public domain. Fact-checkers, while committed to professional journalistic principles, exhibit a distinct identity shaped by epistemological considerations (Mena 2019), as the ultimate goal of their verification efforts is to reach a verdict on the accuracy of claims (Steensen et al. 2023).

Over time, the fact-checking landscape has transformed significantly. It emerged in the 1990s as a means for *political fact-checking*, aimed at holding politicians and other public figures accountable for false statements (Graves and Amazeen 2019). Consequently, fact-checkers were often recognized as “arbiters of political truth” (Graves 2016: 66). In the early 2000s, driven by a “professional reform movement” (Lelo 2022: 1077) in U.S. journalism, fact-checking organizations rapidly expanded globally, with early hubs in Europe and South America, and later growth in Asia, Africa, and the Middle East (Graves and Amazeen 2019). From the early 2010s onward, fact-checking has gained momentum due to changing journalistic standards (Lowrey 2017), technological transformations that have diminished journalists’ gatekeeping role (Graves 2016), and sociopolitical unrest that has sparked demands for accountability (Amazeen 2019). The establishment of the International Fact-Checking Network (IFCN) in 2015 has further globalized the movement (Singer 2021). In response to disinformation campaigns during the 2016 U.S. presidential election, the field’s focus shifted from verifying claims made by politicians to policing viral misinformation on digital platforms—also known as the *debunking turn* (Graves et al. 2023). Here, fact-checkers operate as “gatebouncers” (Cazzamatta and Santos 2023) to “purify” online environments by symbolically removing “uninvited guests” (p. 4).

With the growth of fact-checking as a *global movement*, scholars have observed a “process of institutionalization” (Lowrey 2017: 376) and charted various “areas of convergence” (Graves 2018: 613) with similar organizational structures, standardized practices, and shared challenges (Graves and Lauer 2020; Moreno-Gil et al. 2021). Simultaneously, scholarship has emphasized the diversity within the field, arguing that fact-checkers “from different countries adopt particular styles of fact-checking” (Ferracioli et al. 2022: 719). While previous comparative research has provided notable insights into fact-checkers’ institutional ties (Graves 2018), self-perceptions (Ferracioli et al. 2022), and practices (Humprecht 2020), a more *holistic approach* is needed that brings together usually separate areas of inquiry and allows for a comparative mapping of fact-checking organizations and practices across different contexts. For such systematic and comparative efforts to be both feasible and meaningful, a solid theoretical foundation and clear analytical grid are essential, which is what the concept of *fact-checking cultures*, detailed in the following section, seeks to provide.

## Conceptualizing Fact-Checking Cultures

We define fact-checking culture as the collective set of practices and norms that fact-checkers consciously or unconsciously employ to ensure the veracity of information in the public domain, shaped by the organizational logics within which they operate. In contrast to conceptualizations of fact-checking as *communities of practice* (Brookes and Waller 2023), which draw attention to new occupational identities fostered through collaborative networks, or *deliberate institution-building* (Lauer and Graves 2024), which scrutinizes patterns of influence in an emerging transnational field, the concept of fact-checking cultures broadens the scope and adopts a “culture-as-practice approach” (Knorr Cetina 2007: 364). Drawing on the concepts of *epistemic cultures* (Knorr Cetina 2007) and *journalism cultures* (Hanitzsch 2007), this perspective focuses on the internalized practices and norms of fact-checking, emphasizing the mechanisms of “creating and warranting knowledge” (Knorr Cetina 2007: 363) in the process of distinguishing falsehoods from truths. These internalized practices and norms are intricately intertwined with the organizational environment in which they operate, gradually shaping distinct fact-checking cultures over time. Against the backdrop of the global fact-checking movement, this conceptual angle is productive as it invites inquiries into the similarities and differences in fact-checking that can unfold both within and across organizational and national boundaries.

**Figure 1.**
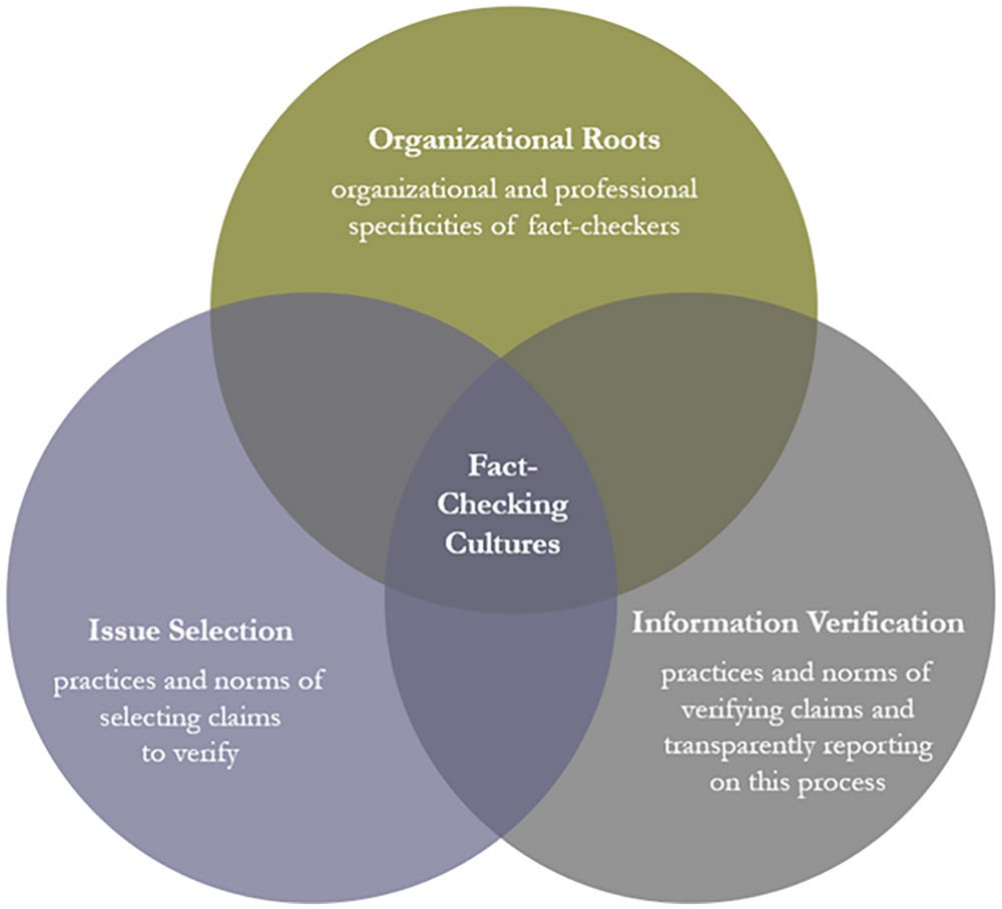
Conceptual framework for studying fact-checking cultures.

With this foundation in mind, we situate and operationalize the concept of fact-checking cultures by proposing a *context-sensitive framework* consisting of three conceptual and analytical components: organizational roots, issue selection, and information verification of fact-checking units (see Figure 1).

### Organizational Roots

The organizational roots of fact-checking entities encompass the organizational and professional specificities of fact-checking organizations, including their affiliation, funding, mission, and the professional backgrounds and role perceptions of fact-checkers. In terms of affiliation, Graves and Cherubini (2016) distinguish between fact-checking entities based in traditional newsrooms and those operating independently. News media-affiliated fact-checking organizations often benefit from a wide audience reach and established infrastructures, but are dependent on financial support and can be influenced by editorial interests. Independent fact-checkers, often partnering with NGOs or universities, enjoy autonomy but may face challenges related to audience reach and resources. Graves’ (2018) mapping of fact-checkers’ ties to the fields of journalism, academia, and politics highlights the hybridity of the landscape and journalists’ willingness to “share jurisdictional authority with non-journalists” (p. 613). Depending on their affiliation, fact-checkers’ missions and role perceptions may differ. While fact-checkers in established newsrooms tend to view fact-checking in “journalistic terms” (Graves and Cherubini 2016: 12) as a duty to inform the public (Brandtzaeg et al. 2018), independent organizations often perceive fact-checking in “activist terms” (Graves and Cherubini 2016: 14), aiming to promote civic engagement (Amazeen 2020). There are, however, regional differences. In the United States and Europe, most fact-checking organizations are affiliated with established media outlets, whereas in non-Western countries, most operate independently, with journalists perceiving their work as a means to counteract political polarization (Lelo 2022). Moreover, organizations outside of Western countries often struggle with insufficient resources, inadequate technology to conduct fact-checking in native languages, and political coercion from local authorities (Hassan et al. 2018; Vinhas and Bastos 2023a). In authoritarian contexts, where the framing of what constitutes misinformation is a matter of “political alignment” (Vinhas and Bastos 2023b: 4), fact-checking can be weaponized as an instrument to silence dissenting voices (Zeng et al. 2017).

Examining the organizational roots of fact-checking entities allows for a better understanding of how fact-checkers select and verify claims. Building on Graves’ (2017) categorization of the core steps of fact-checking—selecting claims, contacting claimants, tracing claims, approaching experts, and reporting on the fact-checking process—a comprehensive picture of fact-checking cultures can be reconstructed.

### Issue Selection

Issue selection refers to the practices and norms of determining which claims are worth checking and which are checkable (Merpert et al. 2018), providing insights into the prevalent issues susceptible to false claims in a given region, as well as the agenda-setting practices of fact-checkers (Vargo et al. 2018).

Identifying misinformation for fact-checking is demanding and time-consuming. Beyond assessing the political and social relevance of claims and the interests of target audiences (Graves 2017), monitoring the (mis)information environment presents fact-checkers with the challenge of avoiding bias and identifying claims that contain verifiable facts (Tsang et al. 2023). In terms of balanced, nonpartisan reporting, Graves and Cherubini (2016) highlight obstacles such as navigating a dynamic political environment, the potential discomfort of fact-checkers scrutinizing their peers, and balancing the selection of prevalent, consequential claims and misinformation from across the political spectrum. As a result, critics have repeatedly accused fact-checkers of harboring editorial biases against conservatives or liberals (Robertson et al. 2020). With respect to identifying checkable claims, fact-checkers are expected to draw a clear line between fact and opinion (Nieminen and Sankari 2021), which can be challenging, especially in times of information scarcity (Graves 2017). Moreover, it is argued that facts can be “ambiguous” (Uscinski and Butler 2013: 162) and that it is not trivial to determine whether statements contain verifiable facts (Merpert et al. 2018).

### Information Verification

Information verification involves the practices and norms of verifying the accuracy of claims—a practice through which fact-checkers ultimately claim epistemic authority. Consequently, they are required to disclose the steps taken and decisions made throughout the process (Graves 2017), allowing readers to draw their own conclusions (Brandtzaeg et al. 2018). While transparency is a key ethical principle for journalists in general, it takes on added significance for fact-checkers, who have “accumulated less public legitimacy” (Kim and Buzzelli 2022: 5) than established news outlets. As emphasized in the IFCN’s code of principles and echoed in scholarship (Humprecht 2020; Kumar 2024), transparency about the fact-checking process is therefore paramount.

The process of information verification involves several steps. First, fact-checkers are faced with the question of whether or not to contact the author of the claim being verified (Graves 2017), with motives for doing so varying, and public figures being easier to locate than (anonymous) social media users. Second, fact-checkers are expected to utilize primary, original evidence, cross-check information from multiple credible sources (Nyhan and Reifler 2012), and provide access to all sources used (Humprecht 2020). In addition, visualizing contextual information can increase the effectiveness of fact-checking (Young et al. 2018). Finally, fact-checkers assign evidence-based verdicts to the verified claims. While most organizations use (visual) scale systems to rate the accuracy of claims (Stencel et al. 2022), others reject such metrics as “unscientific” and “needlessly reductive” (Graves 2018: 626), arguing that claims do not always fit into simple “categories of ‘truth’ or ‘lie’” (Uscinski and Butler 2013: 163). Walter et al. (2020: 368) echo this criticism, arguing that “given the inherent complexities of our political reality, the ratings of statements made by political entities are more likely to be in a shade of gray rather than completely true or false.” However, despite the challenge of assigning narrow labels to verified statements, experimental research shows that such scales increase the accessibility of fact-checks to readers compared to contextual corrections that eschew clear-cut verdicts (Amazeen et al. 2018).

To understand whether and how fact-checkers’ organizational roots, issue selection, and information verification form distinct (trans-)national or (trans-)organizational fact-checking cultures, we exemplify the application and utility of this framework using the fact-checking landscapes of Brazil and Germany as case studies. The following research questions (RQs) guide our analysis:

**RQ1:** What similarities and differences exist among Brazilian and German fact-checkers regarding their *organizational roots*?
**RQ2:** What similarities and differences exist among Brazilian and German fact-checkers regarding their process of *issue selection*?
**RQ3:** What similarities and differences exist among Brazilian and German fact-checkers regarding their process of *information verification*?

## Method

### Country Selection

We chose two comparatively underexplored non-English-speaking nations of global significance (Dias and Sippitt 2020): Brazil, representing the Global South, and Germany, representing the Global North. Both countries share noteworthy similarities and differences facilitating meaningful comparisons. To begin with, Brazil and Germany both have well-established fact-checking landscapes (Stencel et al. 2022), allowing for a nuanced examination of fact-checking cultures at the national and organizational levels. Despite both have democratic political systems—Brazil’s is presidential and Germany’s is parliamentary—their media systems differ considerably. Germany is associated with the democratic-corporatist model, marked by an inclusive media market, high journalistic professionalism, a strong role of the state, and low political parallelism (Humprecht et al. 2022). In contrast, Brazil’s media landscape aligns with the captured liberal model, highlighting the dominance of private commercial media organizations and the state’s limited regulatory capacity (Guerrero 2014), with political parallelism being less relevant due to the country’s decentralized political structure (de Albuquerque 2011). Recent research has demonstrated that these differences in media systems help elucidate commonalities and disparities in national fact-checking endeavors (cf. Cushion et al. 2022; Ferracioli et al. 2022). Hence, the democratic-corporatist model, with its tight connection between the state and the media, could jeopardize the press’s watchdog role due to its dependence on public funding, while the captured liberal model potentially leads to increased scrutiny of politicians by fact-checkers.

### Sample and Data Collection

To identify Brazilian and German fact-checkers, we employed the Google Fact Check Explorer and selected organizations for which at least fifty articles were available. This approach yielded a list of eleven fact-checkers, seven Brazilian and four German (see Table 1). While these organizations do not represent the entirety of fact-checking activity in either country, they are recognized as significant players in the field according to the Duke Reporter’s Lab catalog, the IFCN’s list of verified signatories, and previous research (e.g., Humprecht 2020; Lelo 2022). To explore the diversity of fact-checking cultures, we deliberately avoided limiting our sample to IFCN signatories. Based on this list of fact-checkers, we created *three datasets*.

**Table 1.** Study Sample.

Country	Fact-checker	Fact-checking articles (*N* = 13,498)		Interviewees (*N* = 10)	
				Role	Gender
Brazil	Boatos	2,965	21.97%	Editor	M
Brazil	Aos Fatos	2,798	20.73%	Editor-in-chief	M
Brazil	UOL Confere	1,929	14.29%	—	—
Brazil	Lupa	1,918	14.21%	Reporter	M
Brazil	Estadão Verifica	254	1.88%	Asst. editor	F
Brazil	E-farsas	223	1.65%	Editor	M
Brazil	Fato ou Fake	58	0.43%	—	—
Germany	CORRECTIV.Faktencheck	2,182	16.17%	Editor	F/M
Germany	dpa-factchecking	852	6.31%	Team leader	M
Germany	BR24 #Faktenfuchs	207	1.53%	Editor	F
Germany	ARD-faktenfinder	112	0.83%	Editor	M

First, we consolidated *fact-checkers’ official websites* to gather information about their affiliation, staff, funding models, and mission. Additionally, we reviewed recent application and assessment documents from IFCN signatories for further insights into their organizational structure and methods. These data were utilized to answer **RQ1**.

**Figure 2.**
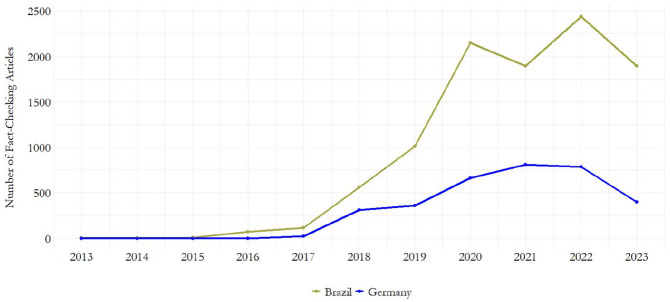
Fact-checking articles over time. *Note*. The sample includes fact-checking articles through July 2023.^a^ ^a^The noticeable rise in articles since 2017 reflects the proliferation of fact-checking organizations beginning in the same year (see Table 2). The spike in 2020 can only be partially attributed to the COVID-19 pandemic, as related fact-checks account for only 15.12 percent of the total dataset (see Supplemental Information file Table C1).

Second, by leveraging the Google Fact Check API and a custom-built scraper, we collected all available *fact-checking articles* from the selected organizations (*N* = 13,498) over eleven years (2013–2023) (see Table 1 and Figure 2). This dataset was employed to address **RQ2** and **RQ3**.

Third, to further enrich and triangulate the findings from the content analyses to answer **RQ1–3**, *qualitative semistructured interviews* were conducted with ten fact-checkers from nine organizations (for details^1^, see Table 1). Potential interviewees were identified by searching the “About Us” sections of fact-checkers’ websites, and at least one representative from each organization was contacted. The final sample consisted of eight editors/reporters, one editor-in-chief, and one team leader. The interviews were conducted between September and November 2023, in Portuguese and German, using a hybrid format that included video calls and written interviews. The video calls, which averaged one hour in length, were recorded, transcribed verbatim, and translated into English. The interview guide covered four main themes: (1) organizational roots and professional background, (2) the selection process, (3) the verification process, and (4) challenges of fact-checking (see Supplemental Information file^2^ Section A).

### Measures and Analyses

This study employs a mixed-methods approach, integrating computational, qualitative, and quantitative content analysis along with qualitative semistructured interviews.

To answer **RQ1**, we conducted a qualitative, in-depth analysis of fact-checkers’ *affiliation* (e.g., independent start-up), *staff background* (e.g., professional journalists), *funding sources* (e.g., advertisement-based), and *mission statement* (e.g., informed public debates) (Graves 2018; Kim and Buzzelli 2022; Singer 2018). During the interviews, we asked fact-checkers about their specific role within the organization, the differences between fact-checking and traditional journalism, and the unique aspects that set their organization apart from other fact-checking initiatives (Amazeen 2019; Cheruiyot and Ferrer-Conill 2018; Lelo 2022). These inquiries provided additional insights into the *organizational and professional specificities* of fact-checking units.

To address **RQ2**, we performed multilingual topic modeling to ascertain the *topic* of the fact-checked claim using the Python library BERTopic version 0.15.0 (Grootendorst 2022). Powered by BERT, this method leverages the capabilities of pretrained transformer language models to generate document embeddings. To embed our multilingual corpus, we utilized the paraphrase-multilingual-MiniLM-L12-v2 sentence-transformers model (Reimers and Gurevych 2019). Uniform Manifold Approximation and Projection (McInnes et al. 2018) was used for dimensionality reduction, followed by hierarchical clustering using HDBSCAN^3^ (McInnes et al. 2017). After excluding outliers from the corpus (2.15%), that is claims that could not be assigned to a specific topic, 110 individual topics were extracted. By examining topic labels and representative articles, we qualitatively merged similar topics, resulting in ninety-seven individual topics and sixteen topic groups. Interviews provided more nuanced insights into the *practices and norms of issue selection*. Accordingly, we asked fact-checkers about their methods for finding claims to fact-check, their selection criteria, and whether they focus on particular domains of public life (Graves 2017; Haque et al. 2020; Micallef et al. 2022).

To answer **RQ3**, we selected a random subset of 550 fact-checking articles—fifty from each organization—and conducted a manual content analysis (for the codebook, see Supplemental Information file Section B). We examined the *accessibility* of the fact-checked claim (0 = no access, 1 = direct access, 2 = indirect access), the *type of verdict* provided in the article (1 = definitive verdict, 2 = narrative verdict), and the *claimant* (e.g., national politician). In addition, we coded whether the article used *visuals* such as infographics (0 = no, 1 = yes) and identified the *sources* cited to support the verdict (e.g., news articles). These variables were either adapted from previous studies (Humprecht 2020; Kim and Buzzelli 2022; Vu et al. 2023) or developed inductively during the preliminary coding phase. The calculation of intercoder reliability for the above-mentioned variables was performed between two coders using a random sample of fifty-five articles (10%), resulting in very good reliability scores (Krippendorff’s α ⩾ 0.80; see Supplemental Information file Section B). To gain deeper insights into the *practices and norms of information verification* and how this process is reported, we asked fact-checkers about their verification methods, whether they contact claimants, what sources they consult, how they decide on their verdict, and what steps they take if claims cannot be reliably verified (Brandtzaeg et al. 2018; Graves 2017; Micallef et al. 2022).

As a core component of our methodological triangulation, we used interview data to contextualize the findings from the aforementioned analyses (**RQ1–3**). The interviews were processed using a qualitative inductive–iterative process (Yom 2015) in which we grouped, condensed, and paraphrased the fact checkers’ responses. Following the steps outlined by Weston et al. (2001), we then inductively identified overarching patterns that emerged from the interviews.

## Findings

The findings section is organized along the RQs and primarily draws comparisons at the country level; for comparisons at the organizational level, see Supplemental Information file Sections C and D.

### Fact-Checkers’ Organizational Roots

**RQ1** assessed the organizational roots of fact-checking entities based on their *affiliation*, *staff background*, *funding*, and *mission*, according to which organizations in Brazil and Germany can be categorized along two axes: news media-affiliated units and independent units (see Table 2).

**Table 2.** Fact-Checkers’ Organizational Roots.

Fact-checker	Launch	IFCN	Affiliation	Staff	Funding	Mission
Estadão Verifica	2018	Yes	Newspaper Estadão (Grupo Estado)	Seventeen employees (professional journalists)	Private media company	“analyze suspicious content that goes viral”
Fato ou Fake	2018	No	Newspaper O Globo (Grupo Globo)	*no information provided*	Private media company	*no information provided*
UOL Confere	2017	Yes	News portal UOL (Grupo Folha)	Two employees (professional journalists)	Private media company	*no information provided*
Aos Fatos	2015	Yes	Independent start-up	Twenty-one employees (multidisciplinary team)	Diversified funding model	“report on the lies of politicians”
Boatos	2013	No	Independent start-up	Two employees (professional journalists)	Advertisement-based funding	“innovate the terms of journalism and Internet technology”
E-farsas	2002	No	Independent start-up	Two employees (system analyst and professional journalist)	Diversified funding model	“using the Internet to demystify the stories circulating on it”
Lupa	2015	Yes	Independent start-up	Thirty-one employees (*no bios available*)	Diversified funding model	“combat misinformation through fact-checking and media education”
CORRECTIV. Faktencheck	2017	Yes	Nonprofit independent newsroom CORRECTIV	Sixteen employees (professional journalists)	Diversified funding model	“lay the foundation for constructive public debate”
BR24 #Faktenfuchs	2017	Yes	Public-service broadcaster Bayerischer Rundfunk (ARD)	Nine employees (professional journalists)	Broadcasting fee	“get to the bottom of rumors”
ARD-faktenfinder	2017	No	News channel tagesschau.de (ARD)	Two employees (professional journalists)	Broadcasting fee	*no information provided*
dpa-factchecking	2013	Yes	German press agency (dpa)	Twenty-five employees (professional journalists)	Private sector	“journalistically advancing the fact-checking format”

*Note*. Data extracted from fact-checkers’ websites, IFCN, and interviews with fact-checkers; missions cited according to statements on websites (accessed: May 2024). IFCN = International Fact-Checking Network.

In Brazil, *news media-affiliated fact-checkers* include Estadão Verifica, Fato ou Fake, and UOL Confere, all launched by large national media conglomerates and operating under the umbrella of their respective news outlets. Consequently, they are primarily funded by these media companies, which, given the historical concentration of media ownership in Brazil, centralize the majority of advertising revenues (de Albuquerque 2019; Lelo 2022). In contrast, German fact-checkers affiliated with news media are housed within public broadcasters and the leading national news agency. BR24 #Faktenfuchs belongs to the public broadcaster Bayerischer Rundfunk, while ARD-faktenfinder is affiliated with the public news channel tagesschau.de, both part of the joint organization of Germany’s regional public-service broadcasters (ARD) and funded by public broadcasting fees. In contrast, dpa-factchecking was established as a unit of the German Press Agency (dpa), a privately organized and financed news agency. As one of the largest fact-checking organizations in Germany, dpa-factchecking emphasizes that it not only checks facts but also promotes fact-checking as a journalistic format. Notably, several independent fact-checkers have significantly larger staffs than those affiliated with media companies. One fact-checker associated with a large media house argued that the extensive coverage provided by the parent organization and the “daily competition with world news” (GER, no. 3^4^) require less verification of facts that are already part of the broader coverage, and therefore fewer human resources.

*Independent fact-checkers* differ significantly from fact-checking operations associated with resource-rich news outlets, particularly in terms of their revenue model. In Brazil, this includes Aos Fatos, Boatos, E-farsas, and Lupa, which were established as independent start-ups, relying on diversified funding sources, such as partnerships with digital platforms (e.g., Meta’s Third-Party Fact-Checking Program), tool development, consulting, donations, or workshops to ensure economic sustainability. These organizations vary widely in staff size (ranging from two to thirty-one employees) and mission. For instance, Aos Fatos aims to “report on the lies of politicians,” while Lupa follows a broader mission of “fighting misinformation through fact-checking and media education.” In contrast, the German sample includes one independent fact-checker, CORRECTIV.Faktencheck, which belongs to the nonprofit newsroom CORRECTIV, and aims to lay “the foundation for a constructive public debate.”

It is important to note that almost all organizations, whether affiliated with news media or independent, share several characteristics: the majority of their fact-checkers are trained as professional journalists, and most of these units are signatories of the IFCN’s code of principles, underscoring their commitment to nonpartisan and transparent fact-checking.

The interviews provided further insights into the *organizational and professional specificities* of fact-checking entities. *First*, fact-checkers acknowledged the constraints of their work, stating that

Disinformation will always be faster than us and, at times, more widespread than we can capture. We also recognize that we cannot convince people already firmly convinced by certain narratives to think otherwise. However, we strive to provide reliable information to those who are uncertain. (GER, no. 4)

*Second*, while fact-checkers saw few differences between traditional journalism and fact-checking practices, they highlighted specific characteristics of their own organization that set them apart from other entities. For instance, one fact-checker emphasized that they “pay special attention to presenting facts in a way that is accessible and understandable even to people with low media literacy” (GER, no. 2). Others stressed that the organization’s long-term goal is to “reduce dependence on funding from major platforms” (BRA, no. 1), or highlighted the organization’s trainee program, which selects journalists from across the country to receive “anti-misinformation training” (BRA, no. 5). Smaller fact-checking organizations emphasized that while they may produce less output, they aim for detailed and in-depth fact-checks (GER, no. 3), “often set the agenda for other media outlets” (BRA, no. 2), and are closer to the community, being treated “as a person rather than a corporation” (BRA, no. 2).

### Fact-Checkers’ Issue Selection

**Figure 3.**
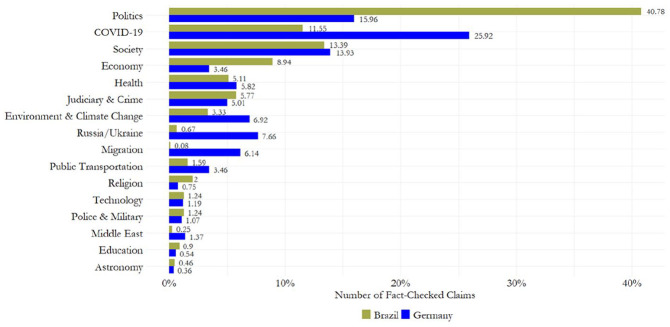
Topic groups of fact-checked claims.

**RQ2** assessed the process of issue selection among Brazilian and German fact-checkers. Figure 3 depicts the *topics of fact-checked claims* aggregated into overarching topic groups. It shows that Brazilian and German fact-checkers covered a wide variety of political, societal, economic, health, environmental, and technological misinformation, among others, with distinctive country-specific emphases. Overall, Brazilian fact-checkers more often verified misinformation from *Politics* (40.78%, compared to 15.96% in Germany), the *Economy* (8.94% vs. 3.46%), *Religion* (2% vs. 0.75%), or *Education* (0.9% vs. 0.54%). In contrast, fact-checking organizations in Germany were more likely to select misinformation related to *COVID-19* (25.92%, compared to 11.55% in Brazil), *Environment & Climate Change* (6.92% vs. 3.33%), *Russia/Ukraine* (7.66% vs. 0.67%), *Migration* (6.14% vs. 0.08%), *Public Transportation* (3.46% vs. 1.59%), and the *Middle East* (1.37% vs. 0.25%).

Country-specific differences are also evident within these topic groups (see Supplemental Information file Section C), particularly with regard to societal issues—a topic group that is almost equally represented in Brazil (13.39%) and Germany (13.93%). While German fact-checkers more frequently reported on misinformation related to *Human Crises and Emergencies* (0.72% vs. 0.15%) and *Protests, Demonstrations, Social Unrest* (5.67% vs. 1.23%), their Brazilian colleagues more often debunked false claims related to the *LGBTQ+ Community* (0.72% vs. 0.06%) and *National Identity and Patriotism* (0.56% vs. 0.09%). Moreover, while the topic of *Human Crises and Emergencies* includes claims with a global scope, such as maritime emergencies or workplace accidents involving illegal migrant workers, misinformation pertaining to the *LGBTQ+ Community* revolves predominantly around national controversies in Brazil. This pattern persists across various topics, underscoring that, beyond verifying misinformation on domestic issues, German fact-checkers tend to select claims linked to global issues more often than their Brazilian counterparts.

Insights from the interviews about the *practices and norms of issue selection* confirm these findings. *First*, German fact-checkers emphasized that, rather than setting topics of interest a priori, they “follow the disinformation” (GER, no. 1) and seek to expand their “focus to foreign countries where state narratives and misinformation are known to be spread” (GER, no. 3). In contrast, their Brazilian colleagues highlighted that “politics is the editorial focus” (BRA, no. 2) and that “topics of national scope” with a domestic focus are given priority (BRA, no. 4). However, others noted that they aim to “diversify the topics of fact-checking” (BRA, no. 3) in order, as one fact-checker explained, to “protect myself against accusations, for example, of bias” (BRA, no. 2). Some fact-checkers working for news media-affiliated organizations emphasized that they are “completely independent” in selecting claims to verify (GER, no. 3), but acknowledged that most misinformation circulates on topics of current interest, which influences the agenda-setting process.

*Second*, all fact-checkers adopt a multiplatform approach to finding claims. Alongside proprietary monitoring tools, they leverage services from platform partners and welcome user suggestions via email or messenger. However, active monitoring of offline events, such as political campaigns, is rare.

*Third*, the criteria for selecting claims are similar in both countries: “We always seek to balance two variables: the reach of disinformation and relevance” (BRA, no. 1). Fact-checking, by definition, excludes “opinion,” “satire,” and “future predictions” (BRA, no. 5; GER, no. 5), and there are restrictions on fact-checking “conspiracy theories” (GER, no. 4) because they usually rely on speculative allegations about groups with hidden agendas, making such claims difficult to verify. Across countries and organizations, interviewees emphasized that “only what can be falsified is verified, even if the result is often more complex than ‘right’ or ‘wrong’” (GER, no. 5).

### Fact-Checkers’ Information Verification

**Figure 4.**
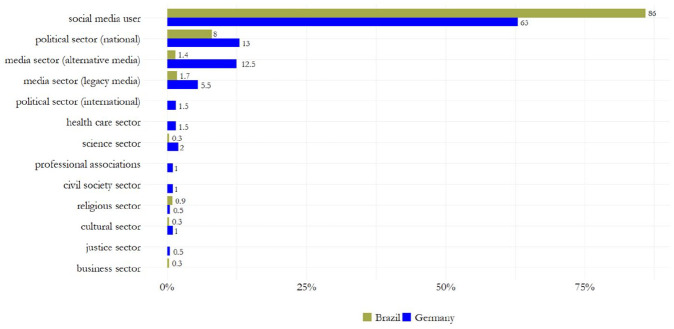
Claimants of misinformation.

**Figure 5.**
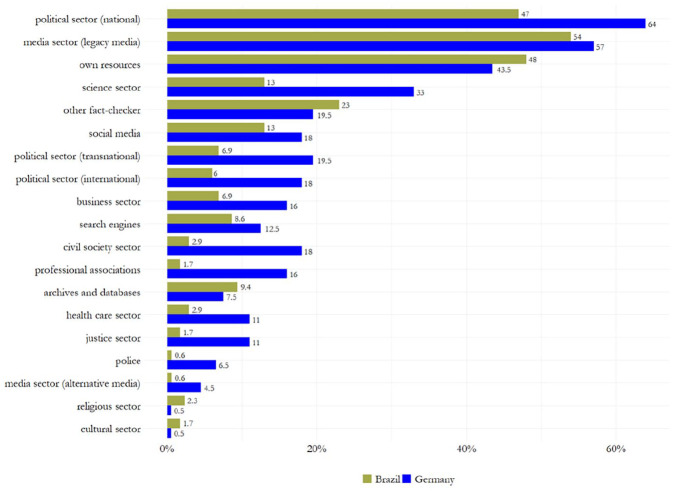
Corrective sources.

**RQ3** investigated the process of information verification among fact-checkers, focusing on the *accessibility* of claims, the *type of verdict*, the *claimant*, as well as *visuals* and *sources* used to support the verdict (see Table 3, Figures 4, 5, and Supplemental Information file Section D). Results show that, first, most fact-checking articles provided *direct access* to the original claim being verified (*N*_GER_ = 124 [62%], *N*_BRA_ = 140 [40%]). Brazilian fact-checkers were more likely to provide indirect access (31.4% vs. 19% in Germany), for example by quoting the verified claim without linking to the original source. Second, the majority of fact-checkers assigned a *definitive verdict* such as “false,” “taken out of context,” or “manipulated” to claims (*N*_BRA_ = 340 [97.1%], *N*_GER_ = 133 [66.5%]). A narrative verdict, which details the results of the fact-check without assigning a clear label, was present in 30 percent of German articles, while it was only rarely used by Brazilian fact-checkers (2.9%). 3.5 percent of German fact-checking articles did not contain any kind of verdict. These articles merely provide contextual information about the topic under discussion, but do not evaluate a specific claim. Third, in Brazil and Germany, fact-checkers most often selected claims made by *social media users* (*N*_BRA_ = 301 [86%], *N*_GER_ = 126 [63%]), followed by public figures or institutions from the national political sector with 13 percent in Germany and 8 percent in Brazil. False claims made by alternative or legacy media were more likely to be verified by German fact-checkers than their Brazilian counterparts, with 12.5 percent versus 1.4 percent and 5.5 percent versus 1.7 percent, respectively. Fourth, German fact-checkers were slightly more likely to include *visuals* such as explanatory videos or infographics in their articles to support the assigned verdict (37.5%) than their fellow fact-checkers from Brazil (30.6%). Finally, with respect to the sources cited to support the verdict, Brazilian and German fact-checkers rely mainly on three types of corrective sources: Sources related to the *national political sector* (*N*_GER_ = 128 [64%], *N*_BRA_ = 164 [47%]), the *legacy media sector* (*N*_GER_ = 114 [57%], *N*_BRA_ = 189 [54%]), and the *fact-checkers’ own resources* (*N*_BRA_ = 168 [48%], *N*_GER_ = 87 [43.5%]), such as previously published articles on related topics. German fact-checkers were more likely to use scientific sources to verify claims (33% vs. 13% in Brazil), while their Brazilian counterparts were slightly more likely to cite articles from other fact-checking organizations (23% vs. 19.5%). Notably, fact-checkers typically cited multiple sources in their fact-checks, with German articles containing a slightly greater variety of sources (*M* = 3.67, *SD* = 1.86) than Brazilian fact-checks (*M* = 2.51, *SD* = 1.20).

**Table 3.** Accessibility, Type of Verdict, and Visuals.

Variable	Brazil (*N* = 350 articles)	Germany (*N* = 200 articles)
Accessibility		
No access	91 (26%)	30 (15%)
Direct	140 (40%)	124 (62%)
Indirect	110 (31.4%)	38 (19%)
Verdict		
No verdict	—	7 (3.5%)
Definitive verdict	340 (97.1%)	133 (66.5%)
Narrative verdict	10 (2.9%)	60 (30%)
Visuals	107 (30.6%)	75 (37.5%)

*Note*. Missing values, such as when a variable could not be determined, are not displayed.

Further similarities and differences in the *practices and norms of information verification* emerged from the interviews. *First*, fact-checkers unanimously stressed the importance of first identifying the originator of the claim and understanding the context, genesis, and distribution of it. Depending on the format of the statement, different verification steps and investigative tools, such as reverse image search or geolocation, are utilized: “It all depends on what we are debunking” (BRA, no. 4). The ultimate goal is to “get as close to the source as possible” (GER, no. 1). A Brazilian fact-checker further emphasized that they “check if the issue has been shared in other languages” to “determine if this misinformation was created here in Brazil or if it was imported from another country” (BRA, no. 3).

*Second*, fact-checkers take different approaches to contacting the person who made the claim. While some typically approach the claimant, others find this rarely productive: “After all, what should we ask this person?” (GER, no. 1). Others only reach out to “public figures” or “accounts that have repeatedly spread false information” (GER, no. 2). Those who choose to contact the claimant argue that it helps them either to “understand the origin of the misinformation” or to “confront the disseminator with the researched facts” and give them the opportunity to respond, which is typically done only with public figures because in the case of lay people, it would be “unnecessary exposure” (BRA, no. 1). Fact-checkers also pointed out that in many cases the claimants cannot be located, inquiries go unanswered, or they are even “made public in order to launch a counterattack” (GER, no. 4). In addition, individuals from the conspiracy theory milieu may use such inquiries to try to “convince” fact-checkers of the validity of the claim (GER, no. 4).

*Third*, the choice of an appropriate verdict reveals another difference among fact-checkers. Although the majority of fact-checking organizations use labels to rate the veracity of a claim, some interviewees acknowledged that reaching a clear verdict is “often not quite straightforward” (GER, no. 1). A fact-checker from Germany argued that “we don’t use these labels because the truth is often not so easy to classify” (GER, no. 4). Others emphasized that “we may publish text without labels if we feel the need to provide context or if available labels don’t meet our needs” (BRA, no. 4). In some cases, “the legal department is even consulted” to come to a decision (GER, no. 5).

*Fourth*, fact-checkers argued that “many claims are often unverifiable” (BRA, no. 1), either because they lack access to trustworthy sources or because information is scarce. In such cases, fact-checkers employ diverse strategies. While some publish a fact-check with the rating “not proven” (GER, no. 1), others pointed out that this “can be very unsatisfactory for readers” (GER, no. 2). Some fact-checkers defer verification “until sufficient new information emerges for a verdict” (BRA, no. 3) or do not publish “content without having a verdict” (BRA, no. 2). However, there are situations in which they deviate from this policy: “In very relevant cases where we know that our audience can help us, we make a publication to indicate that the case is under analysis. Often, we receive very valuable feedback and can reach a verdict.” (BRA, no. 2)

*Fifth*, fact-checkers emphasized high levels of psychological stress due to exposure to violent and harmful content, as well as harassment and personal attacks. However, institutional structures to support fact-checkers in such situations are lacking, especially in smaller units, but deemed urgently needed according to the interviewees. One fact-checker reported receiving psychological support, “but that was my decision” (BRA, no. 3). Larger fact-checking organizations offer support from psychologists or developed coping strategies:

(1) Do not play videos but view individual frames; (2) View pictures/videos in black and white; (3) Turn off the sound; (4) Cover parts of the picture; (5) Review material together whenever possible; (6) Avoid viewing material in the evening whenever possible. (GER, no. 5)

In the case of harassment, which seems to “affect women more than men” (GER, no. 1), a few (again large) organizations offer support to those who experience such attacks, “ranging from digital security training to legal support, with the assistance of the company lawyer” (BRA, no. 1). However, this is considered an exception, leading to a call for cross-organizational support services: “I wish there was an institution that provided some form of legally accessible financial support to reviewers” (BRA, no. 3).

## Discussion

To systematically map fact-checking cultures, this study introduced a *context-sensitive framework* and employed a *cross-national, mixed-methods design*, using Brazil and Germany as case studies. In the following, we will discuss fact-checkers’ organizational roots (**RQ1**), issue selection (**RQ2**), and information verification (**RQ3**) by critically reflecting on areas of divergence and convergence at the country and organizational levels.

### Fact-Checking Cultures: Areas of Divergence and Convergence

At the country level, we find various *areas of divergence*. In Brazil, the fact-checking landscape assembles organizations affiliated with large advertisement-funded media conglomerates, alongside independent entities that rely on diversified revenue models (for similar findings, see Lelo 2022). There, fact-checkers primarily select political misinformation for verification, with an emphasis on national controversies. This finding corroborates Cazzamatta and Santos’s (2023) analysis of fact-checking efforts during the 2022 Brazilian elections, along with Lelo’s (2022) mapping of the Brazilian fact-checking movement, both emphasizing the scrutiny of political statements and campaigns. Remarkably, nearly all verified claims are published with definitive verdicts (cf. Ferracioli et al. 2022; Lelo 2023), and some fact-checkers stressed their overreliance on partnerships with platforms (consistent with Lelo 2022), which serve as vital sources of funding but prevent fact-checkers from holding local politicians accountable due to platform policies (Vinhas and Bastos 2023a). In contrast, the fact-checking landscape in Germany spans organizations affiliated with public broadcasters, the largest national press agency, and an independent newsroom. None of the fact-checking units in our sample have institutional ties to academia or politics (other than Graves’ (2018) cross-national mapping of fact-checkers). Misinformation on a variety of topics, with a focus on both national and international issues, shapes the agenda of German fact-checkers. Compared to their Brazilian counterparts, German organizations select more claims from alternative and legacy media, national and international politicians, or public figures from the cultural and health sectors. Another area of divergence concerns the diversity of corrective sources used to support fact-checkers’ verdict (see also Humprecht 2020), with German organizations utilizing a greater variety of source types per article.

At the organizational level, differences are less consistent. Organizations of various sizes, whether affiliated with news media or operating independently, are verified signatories of the IFCN’s code of principles. However, our analysis of verification practices shows that even small organizations not affiliated with the IFCN adhere to transparent and nonpartisan reporting. For instance, E-farsas, which consists of only two fact-checkers, provides direct access to verified claims and incorporates visual elements for contextualization in most articles. This significantly expands the scope of available scholarship, as the majority of studies focus solely on members of the international network (e.g., Ferracioli et al. 2022; Lelo 2023; for an exception, see Vinhas and Bastos 2023a).

Comparing fact-checking cultures also reveals *areas of convergence* across countries and organizations. Almost all fact-checkers are trained journalists who focus primarily on verifying online misinformation, underscoring the proclaimed debunking turn (Graves et al. 2023) within the field (cf. Cazzamatta and Santos 2023). The digital media environment is arguably a fertile breeding ground for misinformation, and the increased policing of these spaces reflects concerns about information pollution, but may also be driven by commercial partnerships with platform companies. However, the shift toward debunking also raises concerns about the “professional autonomy” of fact-checkers (Graves et al. 2023: 16), as well as the question of missed areas of public life, such as political debates on television. Similarities extend to monitoring strategies, criteria for selecting claims, and steps taken to verify them. Challenges such as high levels of emotional stress and a perceived lack of institutionalized support structures are common across countries and organizations.

Notably, similarities and differences among fact-checkers that do not manifest themselves consistently across organizations and countries (for similar conclusions, see Graves 2018), suggesting that characteristics of national media systems cannot fully explain differences in fact-checkers’ organizational roots, issue selection, and information verification. Consequently, we could not find support for the hypothesis outlined above that, for example, German fact-checkers (democratic-corporatist model) are more reluctant to scrutinize national politicians than their Brazilian counterparts (captured liberal model) because of their financial dependence on public funding; on the contrary. Our results thus also contrast with those of Ferracioli et al. (2022), who show that professional cultures of fact-checkers reflect the characteristics of media systems; it should be noted, however, that the study focused primarily on the watchdog role of fact-checkers and considered only one organization per country, leaving potential differences between organizations in the dark.

We therefore conclude that fact-checking cultures transcend organizational and national boundaries, which might be interpreted as an indicator of *mimetic isomorphism*, where organizations resemble each other and new initiatives emulate and adopt the practices and strategies of established peers, becoming “more like one another due to mimicry, coercion, or common response to a common environment” (Lowrey et al. 2023: 2247). Importantly, however, this mimicry does not come at the expense of diversity within the (trans)national fact-checking landscape, as the identified areas of divergence clearly underscore.

### Limitations

The results of this study must be interpreted in light of its limitations. First, the sample size is confined to two countries and eleven fact-checkers. Although the primary focus of this article is to introduce and operationalize the concept of fact-checking cultures using Brazil and Germany as case studies, a broader range of countries, including varying political systems (e.g., authoritarian vs. democratic regimes) and further media systems (e.g., polarized pluralist model vs. liberal model), could yield a more comprehensive understanding of fact-checking cultures. Future research should also aim to account for more variation within each national fact-checking landscape, for example, by including grassroots organizations or fact-checkers affiliated with academic institutions. Second, our manual content analysis of fact-checkers’ verification practices and interviews with fact-checkers were limited to small samples, which may not allow us to draw strong conclusions about differences among fact-checkers. Future studies should therefore examine a larger sample to capture a broader range of practices, experiences, and challenges in the field.

Other valuable avenues for research include cross-cultural comparisons between fact-checking cultures and journalism cultures (Hanitzsch 2007) to tease out the specificities of each, as well as exploring whether and how the hijacking of professional fact-checking practices by malicious actors, the weaponization of fact-checking, or crowdsourced fact-checks (Allen et al. 2021) might push the boundaries of fact-checking cultures.

## Conclusion

This article illustrates how the framework of fact-checking cultures can aid in comparative analyses of global fact-checking industries. While the existing transnational fact-checking literature is still emerging and often focuses on specific aspects (Ferracioli et al. 2022; Graves 2018; Humprecht 2020), this study underscores a comprehensive approach emphasizing three key components for comparison: fact-checkers’ organizational roots, issue selection, and information verification.

*Conceptually*, this context-sensitive framework enables a holistic examination of fact-checking as collective internalized practices and norms through the lens of “culture” (Hanitzsch 2007; Knorr Cetina 2007). The Brazilian and German case studies illustrated how this framework effectively contextualizes regional fact-checking practices within broader institutional and societal contexts by juxtaposing fact-checkers and identifying their disparities and commonalities. Future research could apply this framework for comparative studies on a larger regional and international scale, which in turn can facilitate the accumulation of comparable evidence for mapping the so-called “global fact-checking” movement. Additionally, the three-dimensional framework could be expanded to include more factors related to organizational structures, professional norms, and the influence of cultural and political elements.

*Methodologically*, the study has demonstrated the utility of a mixed-methods approach to operationalize the concept of fact-checking cultures. The computational approach revealed temporal and topical patterns in the multilingual corpora, while qualitative interviews provide in-depth insights that contextualize quantitative findings within specific social and political settings. This methodological setup could be extended to examine journalistic practices beyond fact-checking.

## Supplemental Material

sj-docx-1-hij-10.1177_19401612241270004 – Supplemental material for “We Follow the Disinformation”: Conceptualizing and Analyzing Fact-Checking Cultures Across Countries Supplemental material, sj-docx-1-hij-10.1177_19401612241270004 for “We Follow the Disinformation”: Conceptualizing and Analyzing Fact-Checking Cultures Across Countries by Daniela Mahl, Jing Zeng, Mike S. Schäfer, Fernando Antonio Egert and Thaiane Oliveira in The International Journal of Press/Politics

## Notes

1. Interviewees explicitly consented to the publication of the details listed in Table 1. Anonymity is still maintained as we refrain from linking (in)direct quotes to individual fact-checkers.

2. The Supplemental Information file is available at the project’s OSF repository: https://osf.io/4mb7g/?view_only=ef622d648ebc462b913f9bfcde3b2994.

3. In HDBSCAN, the min_cluster_size parameter is set to 10, which means that a minimum of ten claims are required to form a topical cluster.

4. The abbreviations *GER* and *BRA* indicate interviews with German and Brazilian fact-checkers, respectively. The number corresponds to the order in which the interviews were conducted.
