# Supplementary material for: “We Follow the Disinformation”: Conceptualizing and Analyzing Fact-Checking Cultures Across Countries
Source: Int J Press Polit. 2024 Aug 11;31(2):264–90. doi: 10.1177/19401612241270004 (PMC12952564; doi:10.1177/19401612241270004)

Supplementary Material for

Mahl, D., Zeng, J., Schäfer, M.S., Egert, F.A., & Oliveira, T. (2024)

“We Follow the Disinformation”: Conceptualizing and Analyzing Fact-Checking Cultures Across Countries

published in *The International Journal of Press/Politics*

Content

[Section A—Interview Guide 3](#_Toc156489321)

[Section B—Codebook for Manual Content Analysis 5](#_Toc156489322)

[Section C—Results Related to the Process of Issue Selection 8](#_Toc156489323)

[Section D—Results Related to the Process of Information Verification 11](#_Toc156489324)

# Section A—Interview Guide

The following questions structured the qualitative interviews with the fact-checkers. Together with computational, qualitative, and quantitative content analyses, they form the basis for answering research questions **RQ1-3**. Depending on the information available on the websites, such as detailed insights into the fact-checking organization’s revenue streams, the selection of questions was tailored to the interviewee. In addition, individual follow-up questions were asked based on the explanations and emphases provided by the fact-checkers.

| **Part I: Organizational Roots and Professional Background** |
| --- |

What is your professional background? How long have you worked as a professional fact-checker? What fact-checking organization(s) do you currently work for?

What is your role at the fact-checking organization?

How is the fact-checking organization you work for funded? What are the primary sources of revenue?

In your opinion, what is the difference between the fact-checking organization(s) you currently work for and other fact-checking initiatives?

What do you think are the main drivers and forces behind the recent rise of fact-checking in your country?

| **Part II: Selecting Claims** |
| --- |

How and where do you find claims to fact-check? What are the typical steps involved?

How do you select claims for fact-checking? What criteria do you use?

Do you have any particular areas/domains of public life that you focus on for fact-checking? Are there areas where misinformation is more prevalent than others? If so, what are they?

| **Part III: Verifying Claims** |
| --- |

Once you have selected a claim for fact-checking, how do you verify its accuracy? What are the typical steps involved?

Do you contact the person who made the claim? If so, why? If not, why not?

What sources do you typically use to verify a claim? Do you consult sources with opposing ideologies or competing views?

Once you have fact-checked a claim, how do you decide what rating or verdict to give it?

If a claim cannot be verified reliably or at all, what do you do?

| **Part IV: Challenges of Fact-Checking** |
| --- |

How do you handle emotionally charged material, such as images and videos of war crimes? Are there any institutionalized structures or support services within your team that fact-checkers can turn to for assistance? If not, what programs or support structures would you like to see?

Have you ever experienced hostility? If so, how do you deal with it? Are there any institutionalized structures or support services within your team that fact-checkers can turn to for assistance? If not, what programs or support structures would you like to see?

What other challenges do you face in the fact-checking process? What do you do when you face these challenges?

# Section B—Codebook for Manual Content Analysis

The following table provides an overview of the variables and categories used in the manual content analysis of fact-checking articles to assess fact-checkers’ information verification practices (**RQ3**), including the intercoder reliability (Krippendorff’s α) based on a random sample of 55 articles (10%) coded by two coders.

**Table B1.** Overview of Variables, Categories, and Intercoder Reliability

| Variable | Category | Intercoder Reliability |
| --- | --- | --- |
| **Accessibility** | **Does the fact-checking article provide access to the original claim being verified, and if so, how?**  0 = no access to the original claim provided  1 = direct access to the original claim (e.g., a link to the original source, such as a YouTube video, or a screenshot of the claim embedded directly in the article)  2 = indirect access to the original claim (e.g., screenshot of unplayable video or audio file; citation of claim without direct link to original source)  (plus, option for open coding) | α = .86 |
| **Verdict** | **What type of verdict does the fact-checking article provide?**  0 = no verdict provided  1 = a definitive verdict (textual or visual) such as “false” or “fabricated” is provided in the title or body of the article  2 = a narrative verdict is provided, usually in the last paragraph or at the top of the article  (*IMPORTANT*: there is no attempt to give a definitive verdict such as “false”)  (plus, option for open coding) | α = .89 |
| **Claimant** | **Who made the fact-checked claim?** The “author” of the fact-checked claim is (part of (the)) …  0 = no claimant mentioned  1 = political sector (national), i.e., national governmental agencies, institutions, organizations, incl. affiliated individuals  2 = political sector (international), i.e., international governmental agencies, institutions, organizations, incl. affiliated individuals  3 = political sector (transnational), i.e., transnational governmental agencies, institutions, organizations, incl. affiliated individuals  4 = media sector (legacy media), i.e., legacy news media, magazines, trade journals, press databases, incl. affiliated individuals  5 = media sector (alternative media), i.e., alternative news media, incl. affiliated individuals  6 = science sector, i.e., scientific institutions, organizations, journals, and publications, incl. affiliated individuals  7 = civil society sector, i.e., civil society organizations and groups, i.e., non-governmental, non-profit groups, or associations of people working for the common good and social issues, incl. affiliated individuals  8 = business sector, i.e., business companies, incl. affiliated individuals  9 = professional associations, i.e., groups that represent professional and political interests, incl. affiliated individuals  10 = religious sector, i.e., churches and religious associations, incl. affiliated individuals  11 = health care sector, i.e., health care institutions, organizations, and facilities, incl. affiliated individuals  12 = cultural sector, i.e., cultural institutions and organizations, incl. affiliated individuals and prominent public figures  13 = justice sector, i.e., institutions and organizations that interpret and apply the law to administer justice, incl. affiliated individuals  14 = social media user(s)  (plus, option for open coding; multiple coding possible) | α = .89 |
| **Visuals** | **Does the fact-checking article provide visuals to support the article’s verdict?**  0 = no  1 = yes | α = .91 |
| **Sources** | **What types of sources does the fact-checking article use to verify the claim and to justify its verdict?** The fact-checking article cites sources from (the) …  0 = no source provided  1 = political sector (national), i.e., national governmental agencies, institutions, organizations, incl. affiliated individuals  2 = political sector (international), i.e., international governmental agencies, institutions, organizations, incl. affiliated individuals  3 = political sector (transnational), i.e., transnational governmental agencies, institutions, organizations, incl. affiliated individuals  4 = media sector (legacy media), i.e., legacy news media, magazines, trade journals, press databases, incl. affiliated individuals  5 = media sector (alternative media), i.e., alternative news media, incl. affiliated individuals  6 = science sector, i.e., scientific institutions, organizations, journals, and publications, incl. affiliated individuals  7 = civil society sector, i.e., civil society organizations and groups, i.e., non-governmental, non-profit groups, or associations of people working for the common good and social issues, incl. affiliated individuals  8 = business sector, i.e., business companies, incl. affiliated individuals  9 = professional associations, i.e., groups that represent professional and political interests, incl. affiliated individuals  10 = religious sector, i.e., churches and religious associations, incl. affiliated individuals  11 = police  12 = archives and databases  13 = search engines  14 = the fact-checking organization’s own resources, incl. affiliated individuals  15 = other fact-checking organizations, incl. affiliated individuals  16 = social media  (plus, option for open coding; multiple coding possible) | α = .80 |

# Section C—Results Related to the Process of Issue Selection

The following table provides a detailed overview of the results of the multilingual topic modeling (BERTopic) used to assess fact checkers’ issue selection practices (**RQ2**), summarizing all 97 individual topics that were manually grouped into 16 overarching topic groups.

**Table C1**. Overview of Topic Groups and Topics

| Topic Groups and Topics | Prevalence (%) | | |
| --- | --- | --- | --- |
|  | **All** (*N* = 13,498) | **Brazil** (*N* = 10,145) | **Germany**  (*N* = 3,353) |
| **Politics** | **34.61** | **40.78** | **15.96** |
| Lula da Silva’s Politics | 6.91 | 9.20 | 0.00 |
| Jair Bolsonaro’s Politics | 5.42 | 7.20 | 0.06 |
| Political Controversies in Brazil | 2.76 | 3.65 | 0.09 |
| Political and Social Affairs | 2.76 | 3.64 | 0.09 |
| Election Fraud and Irregularities | 2.50 | 2.86 | 1.40 |
| Political Controversies in Germany | 2.47 | 0.24 | 9.22 |
| Election-Related Controversies | 1.90 | 2.46 | 0.18 |
| Political Dissent and Polarization | 1.33 | 1.55 | 0.66 |
| Political Protests and Social Movements in Brazil | 1.30 | 1.67 | 0.18 |
| International Relations | 1.16 | 1.36 | 0.57 |
| Government Expenditure | 0.87 | 1.02 | 0.42 |
| Infrastructure Development Under Bolsonaro’s Government | 0.87 | 1.13 | 0.06 |
| Venezuela’s Left-Wing Politics | 0.83 | 1.10 | 0.00 |
| China’s Global Influence | 0.81 | 0.92 | 0.48 |
| Political, Economic, Social Issues in Latin America | 0.66 | 0.88 | 0.00 |
| Bolsonaro and Brazilian Elections | 0.57 | 0.76 | 0.00 |
| 2020 U.S. Presidential Election | 0.41 | 0.33 | 0.66 |
| Politics of the Green Party (Germany) | 0.40 | 0.07 | 1.40 |
| Housing Policies and Regulations | 0.39 | 0.35 | 0.51 |
| Flávio Dino’s Politics | 0.31 | 0.41 | 0.00 |
| **COVID-19** | **15.12** | **11.55** | **25.92** |
| Vaccine Controversy | 6.30 | 4.34 | 12.23 |
| Cures, Prevention, Treatments | 4.02 | 3.67 | 5.10 |
| Origin, Characteristics, Transmission | 1.53 | 1.22 | 2.45 |
| Mask Controversy | 0.90 | 0.48 | 2.15 |
| Home Remedies | 0.59 | 0.64 | 0.45 |
| Statistics and Public Numbers | 0.59 | 0.74 | 0.15 |
| Political Measures | 0.55 | 0.09 | 1.94 |
| Lockdowns and Their Consequences | 0.34 | 0.28 | 0.54 |
| Skepticism About PCR Tests | 0.30 | 0.10 | 0.92 |
| **Society** | **13.52** | **13.39** | **13.93** |
| Public Figures, Social Issues, Controversial Activities | 3.04 | 3.76 | 0.89 |
| Protests, Demonstrations, Social Unrest | 2.33 | 1.23 | 5.67 |
| Sensational News and Mysterious Phenomena | 2.21 | 2.60 | 1.01 |
| Denials and Disputes | 0.83 | 0.85 | 0.78 |
| Manipulation of Public Opinion | 0.77 | 0.53 | 1.49 |
| Conspiracy Theories About Global Organizations | 0.76 | 0.81 | 0.63 |
| Retirement and Pension Controversies | 0.62 | 0.69 | 0.42 |
| Charitable Activities and Assistance Programs | 0.61 | 0.72 | 0.27 |
| LGBTQ+ Community | 0.56 | 0.72 | 0.06 |
| Misattributed Quotes to Public Figures | 0.50 | 0.25 | 1.28 |
| National Identity and Patriotism | 0.44 | 0.56 | 0.09 |
| Bill Gates’ Social Engagement | 0.32 | 0.29 | 0.42 |
| Human Crises and Emergencies | 0.29 | 0.15 | 0.72 |
| Transgender Rights | 0.23 | 0.24 | 0.21 |
| **Economy** | **7.58** | **8.94** | **3.46** |
| Financial Scandals in Brazil | 1.65 | 2.12 | 0.24 |
| Competitions, Gift Promotions, Giveaways | 1.35 | 1.35 | 1.34 |
| Brazil’s Economy | 1.33 | 1.75 | 0.03 |
| Finances and Banking | 0.95 | 1.10 | 0.48 |
| Economic Policies and Labor Issues | 0.74 | 0.92 | 0.21 |
| Promotions, Discounts, Donations | 0.59 | 0.59 | 0.60 |
| Economic Disruptions | 0.59 | 0.62 | 0.48 |
| Job Market | 0.39 | 0.48 | 0.09 |
| **Judiciary & Crime** | **5.58** | **5.77** | **5.01** |
| Deaths and Obituaries | 1.44 | 1.77 | 0.45 |
| Law Enforcement | 1.13 | 1.30 | 0.60 |
| Sexual Violence Against Children | 0.57 | 0.58 | 0.54 |
| Sexual Assault | 0.50 | 0.19 | 1.43 |
| Public Disturbances and Vandalism | 0.47 | 0.58 | 0.15 |
| Criminal Activities | 0.42 | 0.50 | 0.18 |
| Violence and Criminal Acts | 0.42 | 0.34 | 0.69 |
| Child Abductions | 0.36 | 0.20 | 0.86 |
| Criminal Cases and Investigations | 0.26 | 0.31 | 0.12 |
| **Health** | **5.28** | **5.11** | **5.82** |
| Public Health Measures | 1.36 | 1.39 | 1.28 |
| Diseases and Remedies | 1.17 | 1.27 | 0.86 |
| Food, Beverages, Various Substances | 1.11 | 1.31 | 0.51 |
| Healthcare System | 0.72 | 0.30 | 2.00 |
| Reproductive Health and Abortion | 0.47 | 0.39 | 0.69 |
| Cancer Cures and Treatments | 0.45 | 0.44 | 0.48 |
| **Environment & Climate Change** | **4.22** | **3.33** | **6.92** |
| Energy Prices and Taxes | 0.90 | 0.94 | 0.78 |
| Amazon Rainforest Conservation | 0.84 | 1.03 | 0.30 |
| Climate Change Skepticism and Activism | 0.59 | 0.15 | 1.91 |
| Extreme Weather Events | 0.49 | 0.60 | 0.15 |
| Environmental Policies | 0.36 | 0.10 | 1.13 |
| CO2 Emissions | 0.32 | 0.08 | 1.04 |
| Natural Disasters | 0.28 | 0.19 | 0.57 |
| Renewable Energy Technologies | 0.24 | 0.01 | 0.95 |
| Agriculture | 0.21 | 0.25 | 0.09 |
| **Russia/Ukraine** | **2.41** | **0.67** | **7.66** |
| Russian War of Aggression Against Ukraine | 2.06 | 0.66 | 6.29 |
| International Aid for Ukraine | 0.35 | 0.01 | 1.37 |
| **Public Transportation** | **2.05** | **1.59** | **3.46** |
| Automobiles | 1.21 | 0.67 | 2.83 |
| Aviation | 0.64 | 0.64 | 0.63 |
| Motorcycles | 0.21 | 0.28 | 0.00 |
| **Religion** | **1.69** | **2.00** | **0.75** |
| Religious Conspiracy Theories | 0.62 | 0.72 | 0.33 |
| Catholic Church | 0.44 | 0.53 | 0.15 |
| Attacks on Religious Institutions | 0.37 | 0.42 | 0.21 |
| Religious Controversies | 0.26 | 0.33 | 0.06 |
| **Migration** | **1.59** | **0.08** | **6.14** |
| Immigration in Germany and Europe | 1.39 | 0.08 | 5.37 |
| Ukrainian Refugees in Germany and Europe | 0.19 | 0.00 | 0.78 |
| **Technology** | **1.23** | **1.24** | **1.19** |
| Social Media and Surveillance | 0.47 | 0.62 | 0.03 |
| Big Tech Controversies | 0.41 | 0.27 | 0.84 |
| Telecommunications | 0.35 | 0.35 | 0.33 |
| **Police & Military** | **1.20** | **1.24** | **1.07** |
| Militarization and Military Interventions | 0.68 | 0.83 | 0.24 |
| Military Activities and Conflicts | 0.34 | 0.20 | 0.78 |
| Police and Military Operations | 0.18 | 0.22 | 0.06 |
| **Education** | **0.81** | **0.90** | **0.54** |
| Educational System and Politics | 0.60 | 0.67 | 0.39 |
| School Attacks | 0.21 | 0.23 | 0.15 |
| **Middle East** | **0.53** | **0.25** | **1.37** |
| Islam and Muslims | 0.34 | 0.04 | 1.25 |
| Israel and Middle East Relations | 0.19 | 0.21 | 0.12 |
| **Astronomy** | **0.44** | **0.46** | **0.36** |
| Astronomical Phenomena | 0.44 | 0.46 | 0.36 |

# Section D—Results Related to the Process of Information Verification

The following section provides an overview of the manual content analysis used to assess fact-checkers’ information verification practices (**RQ3**).

**Table D1**. Comparing Accessibility, Type of Verdict, and Visual Elements Used

|  | Brazil  (*N* = 350 articles) | | | | | | | Germany  (*N* = 200 articles) | | | |
| --- | --- | --- | --- | --- | --- | --- | --- | --- | --- | --- | --- |
|  | Estadão Verifica | Fato ou Fake | UOL Confere | Aos Fatos | Boatos | E-farsas | Lupa | CORRECTIV.Faktencheck | BR24 #Faktenfuchs | ARD-faktenfinder | dpa-factchecking |
| Accessibility |  |  |  |  |  |  |  |  |  |  |  |
| no access | 2  (4%) | 25  (50%) | 20  (40%) | 2  (4%) | 38 (76%) | 1  (2%) | 3  (6%) | — | 11  (22%) | 19  (38%) | — |
| direct | 10  (20%) | 16  (32%) | 18  (36%) | 31  (62%) | 3  (6%) | 35 (70%) | 27 (54%) | 46  (92%) | 22  (44%) | 10  (20%) | 46  (92%) |
| indirect | 38  (76%) | 5  (10%) | 11  (22%) | 16  (32%) | 5  (10%) | 12 (24%) | 19 (38%) | — | 8  (16%) | 18  (36%) | 3  (6%) |
| Verdict |  |  |  |  |  |  |  |  |  |  |  |
| no verdict | — | — | — | — | — | — | — | — | 4  (8%) | 3  (6%) | — |
| definitive verdict | 50 (100%) | 50  (100%) | 50 (100%) | 50 (100%) | 50 (100%) | 40 (80%) | 50 (100%) | 50  (100%) | 27  (54%) | 29  (58%) | 27  (54%) |
| narrative verdict | — | — | — | — | — | 10 (20%) | — | — | 19  (38%) | 18  (36%) | 23  (46%) |
| Visuals | 15  (30%) | 11  (22%) | 9  (18%) | 23  (46%) | 14 (28%) | 31 (62%) | 4  (8%) | 36  (72%) | 22  (44%) | 16  (32%) | 1  (2%) |

*Note*. *N* = 550 fact-checking articles, 50 for each organization; table reports frequency of coded variables; missing values (e.g., when a variable could not be determined) are not displayed.

**Figure D2**. Comparing Claimants of Misinformation

**
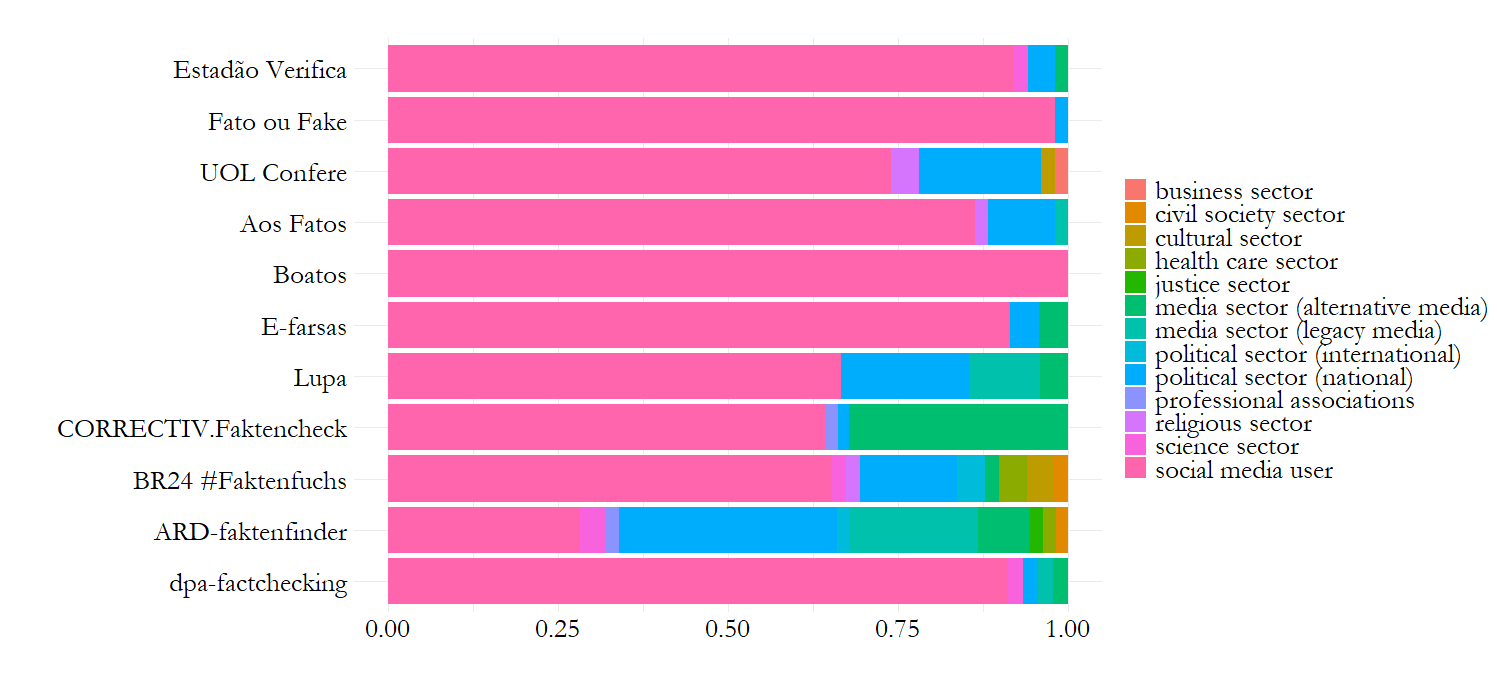
**

**Figure D3**. Comparing Corrective Sources


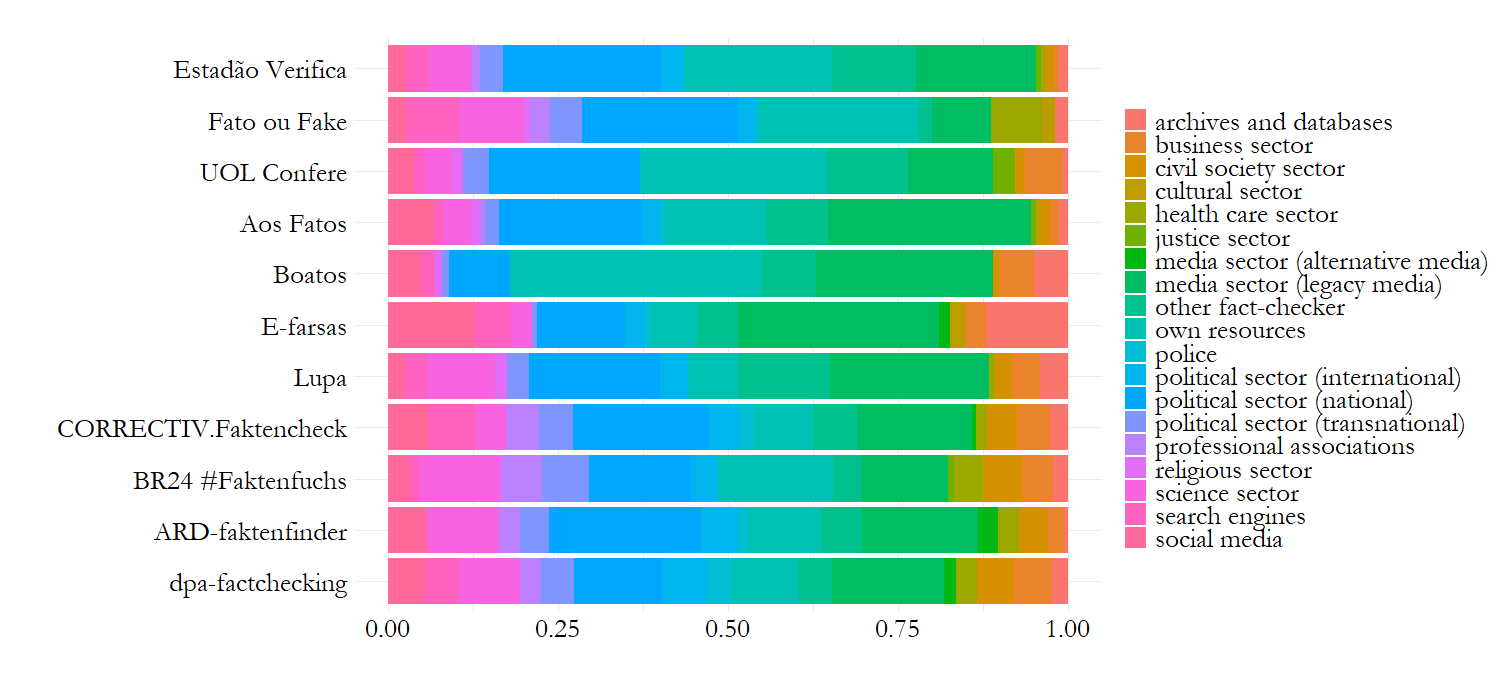

Supplement: sj-docx-1-hij-10.1177_19401612241270004 – Supplemental material for “We Follow the Disinformation”: Conceptualizing and Analyzing Fact-Checking Cultures Across Countries [file sj-docx-1-hij-10.1177_19401612241270004.docx]
